# Noninvasive calculation of the aortic blood pressure waveform from the flow velocity waveform: a proof of concept

**DOI:** 10.1152/ajpheart.00152.2015

**Published:** 2015-07-10

**Authors:** Samuel Vennin, Alexia Mayer, Ye Li, Henry Fok, Brian Clapp, Jordi Alastruey, Phil Chowienczyk

**Affiliations:** ^1^Department of Clinical Pharmacology, King's College London British Heart Foundation Centre, St. Thomas' Hospital, London, United Kingdom;; ^2^Division of Imaging Sciences and Biomedical Engineering, King's College London, St. Thomas' Hospital, London, United Kingdom; and; ^3^Department of Cardiology, Guy's and St. Thomas' Foundation Trust, London, United Kingdom

**Keywords:** central blood pressure, aortic flow velocity, pulse wave velocity, hypertension, left ventricle

## Abstract

Estimation of aortic and left ventricular (LV) pressure usually requires measurements that are difficult to acquire during the imaging required to obtain concurrent LV dimensions essential for determination of LV mechanical properties. We describe a novel method for deriving aortic pressure from the aortic flow velocity. The target pressure waveform is divided into an early systolic upstroke, determined by the water hammer equation, and a diastolic decay equal to that in the peripheral arterial tree, interposed by a late systolic portion described by a second-order polynomial constrained by conditions of continuity and conservation of mean arterial pressure. Pulse wave velocity (PWV, which can be obtained through imaging), mean arterial pressure, diastolic pressure, and diastolic decay are required inputs for the algorithm. The algorithm was tested using *1*) pressure data derived theoretically from prespecified flow waveforms and properties of the arterial tree using a single-tube 1-D model of the arterial tree, and *2*) experimental data acquired from a pressure/Doppler flow velocity transducer placed in the ascending aorta in 18 patients (mean ± SD: age 63 ± 11 yr, aortic BP 136 ± 23/73 ± 13 mmHg) at the time of cardiac catheterization. For experimental data, PWV was calculated from measured pressures/flows, and mean and diastolic pressures and diastolic decay were taken from measured pressure (i.e., were assumed to be known). Pressure reconstructed from measured flow agreed well with theoretical pressure: mean ± SD root mean square (RMS) error 0.7 ± 0.1 mmHg. Similarly, for experimental data, pressure reconstructed from measured flow agreed well with measured pressure (mean RMS error 2.4 ± 1.0 mmHg). First systolic shoulder and systolic peak pressures were also accurately rendered (mean ± SD difference 1.4 ± 2.0 mmHg for peak systolic pressure). This is the first noninvasive derivation of aortic pressure based on fluid dynamics (flow and wave speed) in the aorta itself.

## Abstract (precis)

*For the first time, the entire central aortic pressure waveform is derived from phenomenon occurring in the ascending aorta. This new approach is compatible with current imaging modalities and does not require applanation tonometry or the use of a transfer function*.

## NEW & NOTEWORTHY

*For the first time, the entire central aortic pressure waveform is derived from phenomenon occurring in the ascending aorta. This new approach is compatible with current imaging modalities and does not require applanation tonometry or the use of a transfer function*.

left ventricular (LV) pressure is of key importance in assessing LV performance from pressure-volume relationships and in calculating LV myocardial wall stress. LV wall stress depends both on LV pressure and dimensions (3) and, when elevated, is the major stimulus to adverse LV remodeling, leading eventually to heart failure (20). In the absence of aortic valve disease and a significant gradient across the LV outflow tract, LV pressure during systole approximates central aortic pressure (P) (10). A noninvasive method for estimating central aortic pressure throughout systole coupled to an imaging modality would, therefore, be a uniquely valuable tool in assessment of cardiovascular mechanics.

While there are a number of established noninvasive methods for estimating central systolic pressure (9, 17), these are of limited accuracy, particularly for estimating pressure early in systole at the time of peak myocardial wall stress (9, 22, 24). Second, these methods are difficult to apply during cardiac magnetic resonance (CMR) imaging, the preferred imaging modality for accurate measurement of cardiac dimensions. CMR does, however, provide accurate measures of aortic flow velocity (*U*) and aortic pulse wave velocity (PWV) (7, 34) from which pressure can potentially be estimated during early systole from the water hammer equation (25, 26, 32).

The purpose of the present study was to examine whether aortic pressure can, in principle, be estimated over the whole of the cardiac cycle from noninvasive measures of *U*, PWV, mean arterial blood pressure (MAP), diastolic blood pressure (DBP), and the diastolic decay of pressure. The last three are similar at peripheral and central sites (27, 35) and can be measured noninvasively during imaging. We formulated a part theoretical, part empirical algorithm to determine pressure from flow, peripheral blood pressure, and a single parameter of the artery tree: local aortic PWV. We tested this first using a numerical model to calculate pressure from flow according to fluid dynamic principles for given dimensions and elasticity of the whole arterial tree. This approach has the advantage that it tests the optimal performance of the algorithm when there is no experimental error in determining local PWV, or in the measurement of pressure or flow. We then tested the algorithm using clinical data in which aortic root pressures and flow velocities were measured using a combined pressure/Doppler flow transducer at the time of cardiac catheterization.

## METHODS

### 

#### Algorithm to estimate aortic pressure from aortic flow velocity.

**Fig. 1.**
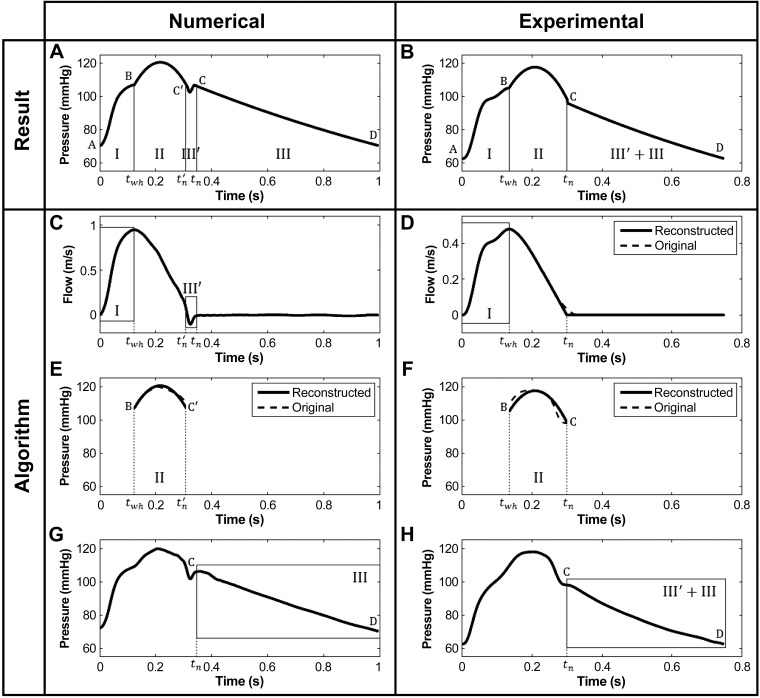
Nomenclature and description of the algorithm for reconstructing the central aortic pressure waveform (*top*). This waveform, referred to as P, is divided into 4 parts if the flow velocity (*U*) contains a region of reverse flow (*A, C, E, G*) or 3 parts if *U* does not reverse (*B, D, F, H*). P is determined by combining the water hammer (parts I and III′) with the diastolic (part III) pressure waveforms and a second-order polynomial (part II).

The principle of the algorithm is that the aortic pressure (P) waveform is entirely reconstructed from aortic flow velocity waveform, PWV, and blood pressure components: DBP, MAP, and diastolic decay. The target waveform is divided into 4 parts (Fig. 1) defined by characteristics of the flow velocity (*U*) waveform. Hemodynamic principles of continuity of pressure, the relationship between P and *U*, in early systole and conservations of MAP, DBP, and diastolic decay along the arterial tree are then used to determine P as detailed below.

In early systole (part I), P*(t)* can be calculated using the water hammer equation (19, 26) under the assumption of negligible wave reflections. Hence, the relationship between P and *U* is assumed to be linear and given by:

(1) $$\text{P}\;={\text{P}}_{\text{wh}}=\rho \cdot \text{PWV}\cdot U$$

with P_wh_ the water hammer pressure and ρ = 1,060 kg/m^3^ the density of blood (which is assumed to be constant). Moreover, P(*t = 0*) in part I is set equal to DBP (*point A*, Fig. 1*A*) and the end of part I is defined as the time of peak flow velocity (*point B*, Fig. 1, *A* and *C*). PWV is calculated from changes in aortic blood pressure (dP) and flow velocity (d*U*) by the sum-of-squares method (12) originally derived for the coronary arteries:

(2) $$\text{PWV}=\frac{\text{1}}{\rho }\sqrt{\frac{\sum {\left(\text{d}P\right)}^{\text{2}}}{{\left(\text{d}U\right)}^{2}}}$$

where the sum is taken over the whole cardiac cycle.

Part III of P is determined as follows: the exponential decay in the measured diastolic pressure waveform is transformed, using a logarithm function, into a linear spline whose coefficients are computed by using the polyfit function in Matlab (The MathWorks, Natick, MA) based on linear least-squares fitting. The inverse of the coefficient of the linear spline is taken as the time constant (τ) of the exponential decay in the diastolic part of the pressure waveform as follows from *Eqs. 3* and *4*:

(3) $$\text{P}\left(t\right)={\text{P}}_{\text{0}}\cdot e^{\frac{-t}{\tau }}$$

(4) $$\text{In}\left[\text{P}\left(t\right)\right]=\text{In}\left({\text{P}}_{\text{0}}\right)-\frac{\text{1}}{\tau }\cdot \text{In}\left(t\right)$$

where P_0_ is the pressure at *t* = *t*_n_.

The calculated P_wh_ is constrained to end at DBP (*point D*, Fig. 1, *A* and *G*), while starting at *point C*, the end of the dicrotic notch where the flow velocity is approximately zero after the closure of the aortic valve. The pressure asymptote was assumed to be zero.

The dicrotic notch is present in P_wh_, calculated using *Eq. 1*, if *U* contains a region of reverse flow during the closure of the aortic valve at the end of systole. In that case, the notch is incorporated into the reconstructed waveform P, starting at *point C*′ and ending at *point C* (part III′, Fig. 1, *A* and *C*). *Point C*′ is located on the exponential curve extended from part III to part III′ at the time of the first zero crossing of *U* (*t*_n_′, Fig. 1*C*).

Part II, which encompasses the systolic peak, is approximated by a second-order polynomial: *y = at*^2^ *+ bt + c*, between the time of the water hammer pressure peak (*t*_wh_) and estimated notch (*t*_n_′) (Fig. 1*E*), the times when part II is connected to parts I and III′ + III. This polynomial passes through *points B* and *C*′ (Fig. 1, *A* and *E*). A system of three equations is needed to obtain the three constants *a, b*, and *c*:

(5) $$\{\begin{array}{c} at_{\text{wh}}^{\text{2}}+bt_{\text{wh}}+c={\text{P}}_{\text{ao}}\left(t_{wh}\right)\\ at_{\text{n}}^{\text{2}}+bt_{\text{n}}+c={\text{P}}_{\text{ao}}\left(t_{\text{n}}\right)\\ \frac{\text{1}}{T_{\text{2}}}\int_{t_{\text{wh}}}^{t_{\text{n}}}\left(ax^{\text{2}}+bx+c\right)\cdot dx=M_{2} \end{array}$$

(6) $$M_{\text{2}}=\frac{\text{MAP}\cdot T-M_{\text{1}}\cdot T_{\text{1}}-M_{\text{3}}\cdot T_{\text{3}}}{T_{\text{2}}}$$

**Fig. 2.**
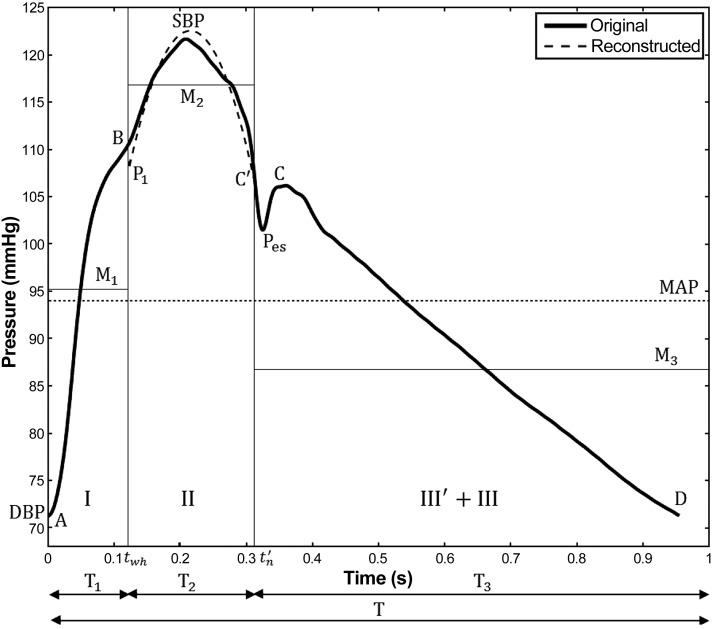
Nomenclature for the calculation of part II of the reconstructed aortic pressure waveform. Midsystole is approximated as a second-order polynomial that satisfies continuity and produces the prescribed mean arterial pressure (MAP). SBP, systolic blood pressure; DBP, diastolic blood pressure.

The first two equations are obtained by imposing the following continuity conditions at *t*_wh_ and *t*_*n*_, when the reconstructed pressure P has to be equal to P(*t*_wh_) and P(*t*_n_), respectively. The last equation of system *Eq. 5* is found by first equating MAP to the time-weighted average of the mean pressures of part II (*M*_2_) and parts I and III′ + III (*M*_1_ and *M*_3_, respectively) taking into account the duration of parts I (*T*_1_), II (*T*_2_), and III′ + III (*T*_3_) (see *Eq. 6* and Fig. 2). The last equation in *Eq. 5* is then obtained by imposing the condition that the mean of the area under the curve must be equal to *M*_2_.

#### Testing the algorithm using numerically derived pressure and flow waveforms.

To test the algorithm (described above in *Algorithm to estimate aortic pressure from aortic flow velocity*) under optimal conditions in which the input local PWV and pressure and flow waveforms were prespecified or derived theoretically with zero experimental error, we used a previously validated, nonlinear, single-tube, one-dimensional model of the arterial tree (2). The properties of this model are described in table II of Ref. 2. This model generates the theoretical pressure corresponding to a prespecified flow input from the parameters (dimension and elasticity) of the tube, including local PWV, and thus provides a theoretical test of the algorithm. Fifteen samples were generated where the compliance *C* was set at 10.16 mm^3^/Pa and PWV was varied from 3.55 to 7.09 m/s. The process was repeated for 12 samples with PWV equal to 5.02 m/s, and with *C* varying between 6.1 and 28.46 mm^3^/Pa.

#### Testing the algorithm using clinical data: measured pressure and flow waveforms.

Simultaneous invasive recordings of aortic pressure and flow velocity were available in 18 patients (mean ± SD: age 63 ± 11 yr, aortic BP 136 ± 23/73 ± 13 mmHg) in whom a combined solid state pressure sensor and Doppler ultrasound probe (Combowire XT, Volcano, Rancho Cordova, CA) was used to record pressure and flow velocity in the aortic root. Each dataset consisted of at least 10 cardiac cycles that were first filtered using a Butterworth low-pass filter that has been shown to eliminate noise without influencing waveform characteristics (16) and then ensemble averaged using the foot of the waveform as fiducial time to produce a single waveform.

**Fig. 3.**
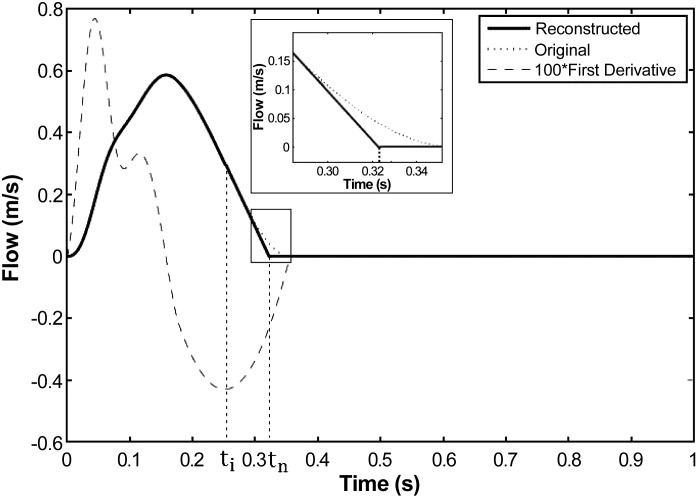
Extrapolation of the connecting point *t*_n_ in experimental data in absence of the dicrotic notch. The slope of the inflexion point *t*_i_ is prolonged until the flow is null.

Flow velocity recordings did not show any region of reverse flow during the closure of the aortic valve, while the dicrotic inflexion was present in all pressure waveforms. We therefore modified the four-part calculation of P(*t*) described above (in *Algorithm to estimate aortic pressure from aortic flow velocity*) by connecting parts II and III directly (Fig. 1, *right*). The inflexion point at *t*_i_ was defined as the minimum of the first derivative of the *U*(*t*) waveform (Fig. 3). On the *U* waveform, the slope at *t*_*i*_ was then extrapolated to zero at *t*_*n*_ (*inset* plot in Fig. 3). The resulting three-part model still uses part I and the new part III + III′ to estimate part II from MAP and enforcing continuity of pressure through *points B* and *C* (Fig. 1, *B* and *F*).

#### Error calculation.

Each dataset from the numerical model comprised pressure waveforms theoretically derived from the flow waveform for a given set of model parameters (with either varying *C* or PWV). The pressure reconstructed from the simplified algorithm was then compared with the “reference” pressure derived using the full fluid dynamic model by means of the root mean square error (RMSE). This error is given by:

(7) $$\text{RMSE}=\sqrt{\frac{\sum_{1}^{N}{\left({\text{P}}_{i}^{\text{reconstructed}}-{\text{P}}_{i}^{\text{reference}}\right)}^{2}}{N}},$$

where *N* is the number of points in each sample, P^reconstructed^ is the pressure computed by the algorithm and P^reference^ is the target pressure. The results presented in the tables are the means ± SD RMSE for all samples.

Additionally, absolute errors between characteristic points on reconstructed and reference waveforms, such as the first systolic shoulder (P_*1*_, *point B*, Fig. 1, *A* and *B*), systolic peak pressure (SBP), and end-systolic pressure (P_es_, *point C*, Fig. 1, *A* and *B*), were expressed as the mean ± SD difference between reconstructed pressure and reference pressure at these points.

A similar approach was taken to compare pressure reconstructed from experimentally measured aortic flow velocity and the experimentally measured reference aortic pressure. The RMSE was calculated as a mean for all subjects.

## RESULTS

### 

#### Numerical data.

**Fig. 4.**
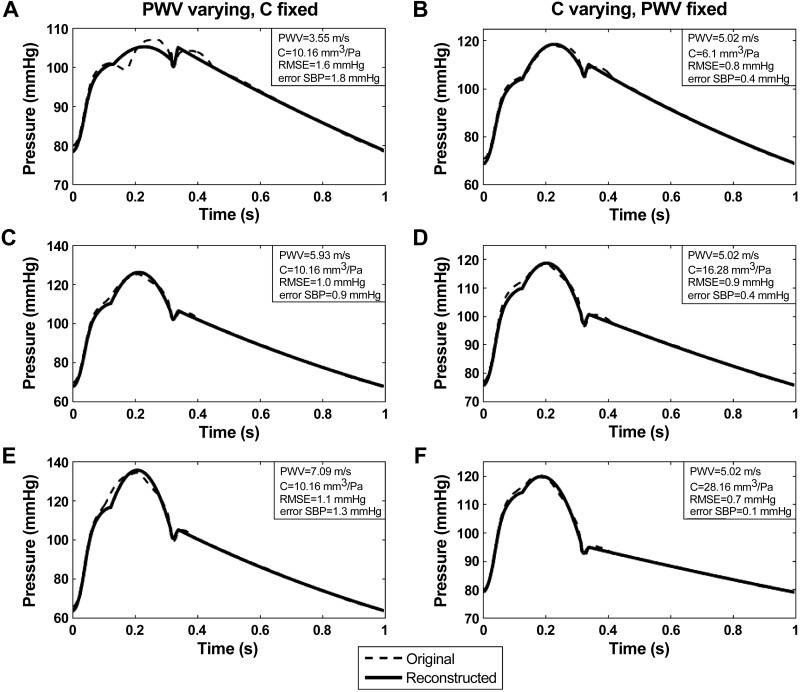
Comparison between reconstructed pressure (continuous line) and numerically-generated reference pressure (dashed line) waveforms. For the left panels (*A, C, E*), the compliance *C* is fixed at 10.16 mm^3^/Pa and PWV is varying. On the right (*B, D, F*), PWV is set at 5.02 m/s and *C* is changing. RMSE, room mean square error.

For numerical data, pressure waveforms reconstructed from flow velocities closely resembled reference pressures with a mean (across all samples obtained with both sets of model parameters) RMSE for the whole waveform ≤ 2 mmHg (Fig. 4, Table 1). Similarly, the mean error for each of the characteristic points (P_*1*_, SBP, P_es_) was ≤2 mmHg and the maximum error for any sample was smaller than 2 mmHg for SBP and P_es_, and 6 mmHg for P_*1*_ (Fig. 2, Table 1). The main difference between simulated and reconstructed waveforms arose after the first systolic shoulder. The notch was well captured in the results shown in Fig. 4, but the algorithm does not render the gap in midsystole for small values of PWV (Fig. 4*A*). Finally, the approximation of the second part of systole by a second-order polynomial yielded estimates of SBP with a mean error ≤2 mmHg.

**Table 1.** Summary of errors between aortic pressure estimated from flow velocity and reference pressure for numerically simulated data

			Waveform	P_1_		SBP		P_es_	
No. of Datasets	PWV, m/s	*C*, mm^3^/Pa	Mean ± SD RMSE, mmHg	Mean ± SD, mmHg	Max, mmHg	Mean ± SD, mmHg	Max, mmHg	Mean ± SD, mmHg	Max, mmHg
15	3.55–7.09	10.16	1.1 ± 0.2	1.7 ± 2.9	5.7	0.5 ± 0.9	1.8	1.7 ± 0.1	1.8
12	5.02	6.10–28.16	0.9 ± 0.1	0.3 ± 1.1	2.4	0.3 ± 0.3	0.5	1.4 ± 0.4	2.1

PWV, pulse wave velocity; *C*, compliance; RMSE, root mean square error; P_1_, first systolic shoulder; SBP, peak systolic aortic pressure; P_es_, end-systolic shoulder; Max, maximum value of error.

#### Experimental data.

**Fig. 5.**
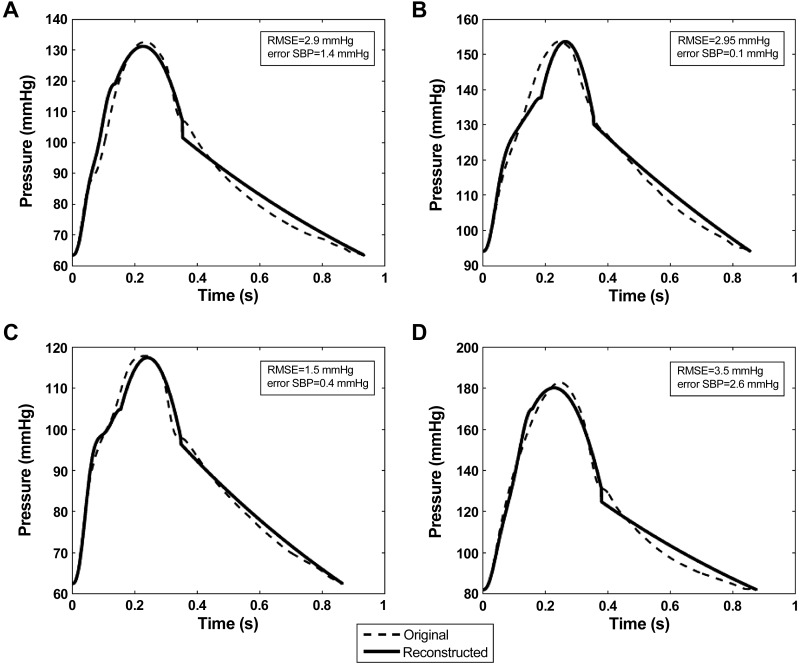
*A–D*: comparison between aortic pressure reconstructed from a Doppler flow velocity transducer in the ascending aorta (dashed line) and measured pressure at the same point (continuous line).

**Fig. 6.**
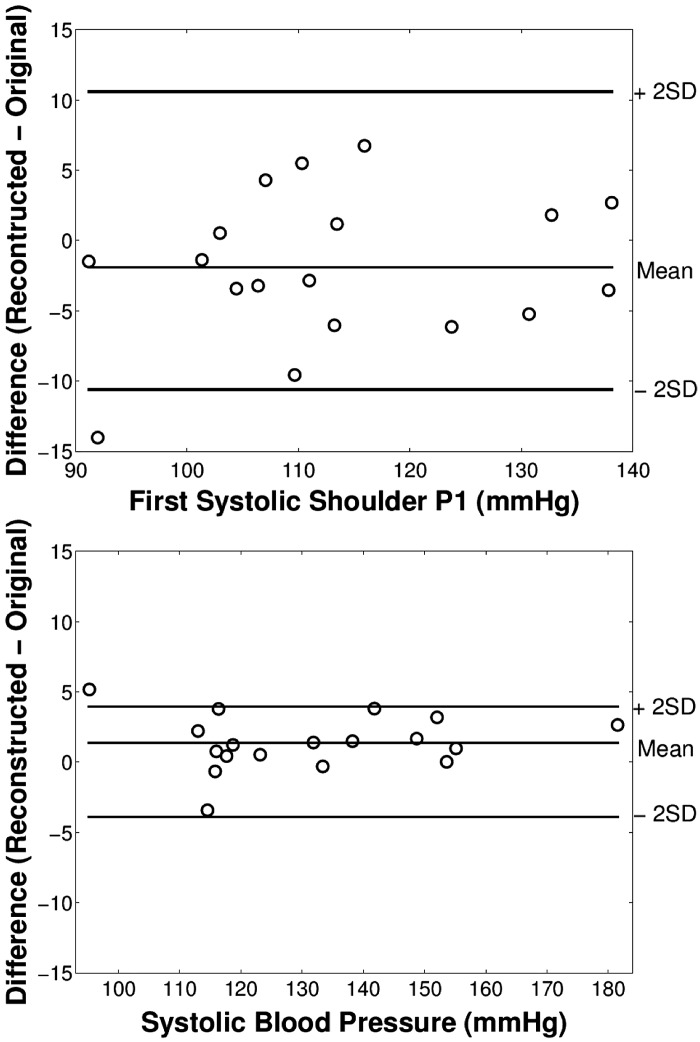
Modified Bland-Altman plots (with the abscissa replaced by reference pressures) for difference between pressures estimated from measured flow velocity and measured pressure for first systolic P1 (*top*) and peak aortic SBP (*bottom*) pressures.

The characteristics of the subjects studied are shown in Table 2. For measured aortic pressure and flow velocities, pressure waveforms reconstructed from flow velocities closely resembled reference pressures (Fig. 5). The mean RMSE for the whole waveform for all subjects was 3.4 mmHg (Table 3). For P_*1*_, SBP, and P_es_, mean errors for all subjects were ≤2 mmHg and the maximum error was 14 mmHg (Table 3) with differences mainly occurring in diastole (Fig. 5). The maximum error arose in the subject with the greatest variation in the decreasing phase of the flow velocity leading to a poor estimation of the time of valve closure. Standard deviations for errors of P_*1*_ and SBP were, respectively, 5.3 and 2.0 mmHg (Table 3 and Fig. 6).

**Table 2.** Demographics for participants used in the experimental study

Characteristics	Mean ± SD or *n* (%)
Age, yr	61.8 ± 10.3
Male	9 (50%)
BMI, kg/m^2^	28.3 ± 6.3
SBP, mmHg	136 ± 23
DBP, mmHg	73 ± 13
Medical history	
Smoker	2 (11%)
Diabetes	5 (28%)
Myocardial infarction	7 (39%)
PCI/CABG	7 (39%)
CAD	
Single vessel	7 (39%)
Two vessels	4 (22%)
Three vessels	3 (17%)
Drug therapy	
Nitrate	4 (22%)
Beta blocker	7 (39%)
ACEI/ARB	9 (50%)
Diuretic	0 (0%)
CCB	5 (28%)

SBP, systolic blood pressure; DBP, diastolic blood pressure; BMI = body mass index; PCI, percutaneous coronary intervention; CABG, coronary artery bypass graft; CAD, coronary artery disease; ACEI, angiotensin-converting enzyme inhibitor; ARB, angiotensin receptor blocker; CCB, calcium channel blocker.

**Table 3.** Summary of errors between the aortic pressure reconstructed from aortic flow velocity measured in the ascending aorta and measured aortic pressure

Waveform	P_1_		SBP		P_es_	
Mean RMSE + SD, mmHg	Mean ± SD, mmHg	Max, mmHg	Mean ± SD, mmHg	Max, mmHg	Mean ± SD, mmHg	Max, mmHg
3.4 ± 1.3	1.9 ± 5.3	14	1.4 ± 2.0	5.2	0.9 ± 3.4	6.9

SBP, peak systolic aortic pressure; Max, maximum value of error.

## DISCUSSION

The novel finding of the present work is that, in principle, the entire aortic pressure waveform can be generated from the aortic flow velocity waveform, local PWV, and peripheral BP components (diastolic decay, diastolic and mean blood pressures). Our numerical simulations and testing against clinical data show that, except at very low values of PWV, pressure can be estimated to within a few millimeters Hg, an error that is small in comparison with that in the measurement of peripheral BP [typically in the range of 10/5 mmHg from true intra-arterial BP (28)]. Although errors for experimental data were within acceptable limits, they were higher than those obtained from numerical data. This probably reflects some experimental error in acquisition of the pressure and, particularly, the flow data which is sensitive to transducer orientation.

For peak systolic blood pressure these errors are also small in comparison with the error typically ranging from 10 mmHg in older subjects (18) up to 20 mmHg in children and young adults (23) if the amplification between aortic and peripheral upper limb blood pressure is ignored (i.e., aortic SBP is assumed equal to brachial or radial SBP). Although pressure wave reflection early in systole might be expected to distort the relationship between pressure and flow, we found no evidence of this since errors in pressure estimation from the water hammer equation were minimal. This is consistent with recent experimental observations that pressure reflection may be less important than previously thought (14).

There are several potential advantages of our new method: it may be the only practical method of obtaining the aortic waveform during CMR when transfer function approaches based on carotid or radial tonometry are not possible. A transfer function approach based upon cuff blood pressure has been introduced recently (6, 33) and could potentially be used during CMR but, although such transfer functions are reasonably accurate (within limitations imposed by peripheral BP calibration), for the estimation of peak central aortic systolic pressure, they are less accurate in estimating high-frequency components of the waveform, such as the first systolic shoulder P_*1*_ (9, 22, 24). This is particularly important for the assessment of peak myocardial wall stress, which is usually coincident with the first systolic shoulder (11) and thus directly related to P_*1*_. The method also provides a means of estimating peak central aortic blood pressure using personalized hemodynamic profiling without the assumptions inherent in a generalized transfer function.

From a physiological standpoint, the present work shows that pulsatile components of BP are determined almost exclusively from PWV and aortic flow velocity. Pulsatile components of both peripheral and central BP increase more with age than do MAP and DBP (8, 15) and are important determinants of cardiovascular risks (30), as is aortic PWV (31). Since the pulsatile BP components and PWV are linked through aortic flow velocity, aortic flow velocity may also be an important determinant of cardiovascular risk and deserves further evaluation in this regard. Furthermore, measuring PWV and flow velocity profiles should allow pulsatile components of pressure to be partitioned into those that arise purely as a result of aortic stiffening and those that arise through altered ventricular dynamics and hence flow velocity.

It is important to note that the results we have obtained provide only a “proof of principle,” since we have assumed that PWV and peripheral BP components (MAP, DBP, and the diastolic decay) that are invariant between central and peripheral arteries can be measured with no significant error compared with their derivation from the simulated or measured intra-aortic pressure. Dependence on measurement of MAP and DBP is common to all methods of estimating central aortic BP. While not conventionally derived during oscillometric BP measurement, the diastolic decay can be estimated from a cuff waveform. Furthermore, its importance is restricted to estimating the aortic waveform during diastole which for the purposes of assessing LV-vascular coupling is of limited value, since closure of aortic valve decouples aortic and LV pressure. Thus the major additional limitation of our proposed method, compared with existing methods, relates to the accuracy with which local aortic PWV can be measured. PWV has a major impact on calculation of aortic pressure during early systole since the water hammer pressure at that time is the product of flow velocity and PWV (*Eq. 1*). Thus any errors in PWV are transferred directly to the calculated aortic pressure. For low values of PWV, water hammer pressure may start to decay early in systole leading to an oscillation in pressure (Fig. 4*A*) which is not completely captured in our fitting of midsystole. However, this only occurs at low values of PWV unlikely to be encountered in adults.

There are several methods for assessing aortic PWV using CMR (or ultrasound imaging). Local PWV can be assessed from the relation between aortic distension and flow velocity (13) or area (A) and flow velocity (4, 21) using similar assumptions as those we used in the present proof of concept, where in early systole the relationship between P and *U* is assumed to be linear. The so called P-*U* loop methods make the same assumptions. The wave speed is then obtained from the coefficient of proportionality between these two measures (21). Early local reflections may result in an overestimation of local PWV by the P-*U* method but underestimation by the *U*-A method (local reflections distending the artery relative to flow) and therefore using a combination of both methods may be advantageous (29). Alternatively, PWV can be determined from the foot-to-foot method—the time delay between arrival of the aortic velocity (or aortic distension) waveform between two sites in the aorta (typically the ascending arch and diaphragm) (5). This method gives an integrated measure over the pathway between the two sites and, hence, may differ from the local PWV, and the exact sites chosen may be important. Using two closely separated sites (e.g., ascending and descending arch) may give a value of PWV more closely related to local PWV, but has the disadvantage that it requires high-temporal resolution. Wentland et al. (34) have shown data that interleaving in Fourier velocity-encoded (FVE) M-mode imaging provides better temporal resolution and may allow local PWV to be measured along a relatively short segment of the aorta. Further experimental work will be required to determine the best method for noninvasive assessment of PWV for the purposes of prediction of aortic pressure from flow. Because it is difficult to measure aortic pressure invasively during CMR, this will likely require a validation in a relatively large number of subjects with CMR and invasive pressure measurements performed on two separate occasions, with correction for any differences in BP between the two occasions. A further limitation of our work is that our clinical data were restricted to a relatively small sample of middle-aged to older subjects. Further work will be required to validate the method in a larger sample. In particular, the algorithm may not describe the secondary “bump” often seen in younger people around the dicrotic notch which may be due to an early diastolic reflected wave, nor waveform changes resulting from valve lesions or congenital heart disease. It is possible that a more complex model is required to capture the full pathophysiological range of waveform morphologies and to include subtle features such as those related to valve leaflet motion (1).

In conclusion, we have shown that the central ascending aorta pressure waveform can be accurately predicted from aortic flow velocity, local aortic PWV, and space-invariant components of BP—MAP, DBP, and diastolic BP decay—measures that can be readily derived during CMR. Unlike existing methods for estimating central aortic blood pressure, the present approach provides aortic pressure over the whole cardiac cycle, does not require carotid or radial tonometry, makes no assumptions regarding generalizability of a transfer function, and has the potential to improve the accuracy with which early systolic components of BP that determine peak myocardial wall stress can be determined. Further validation is required to quantify the influence of errors introduced by measurement of local aortic PWV and BP components.

## GRANTS

This work was supported by a British Heart Foundation Research Excellence Award RE/08/003 Interdisciplinary PhD Studentship to S. Vennin. J. Alastruey gratefully acknowledges the support of an EPSRC project grant (EP/K031546/1), a British Heart Foundation Intermediate Basic Science Research Fellowship (FS/09/030/27812), and the Centre of Excellence in Medical Engineering funded by the Wellcome Trust and EPSRC under Grant Number WT 088641/Z/09/Z. The authors acknowledge support from the National Institute for Health Research (NIHR) Clinical Research Facility at Guy's & St Thomas' NHS Foundation Trust and NIHR Biomedical Research Centre based at Guy's and St Thomas' NHS Foundation Trust and King's College London. The research leading to these results has also received funding from the EU FP7 for research, technological development, and demonstration under grant agreement VP2HF (no. 611823).

## DISCLOSURES

P. Chowienczyk has an interest in Centron Diagnostics, a King's College London spin-out company formed to exploit technology in blood pressure measurement.

## AUTHOR CONTRIBUTIONS

Author contributions: S.V., A.M., Y.L., H.F., J.A., and P.C. conception and design of research; S.V. analyzed data; S.V., J.A., and P.C. interpreted results of experiments; S.V. prepared figures; S.V. drafted manuscript; S.V. and J.A. edited and revised manuscript; B.C. performed experiments; J.A. and P.C. approved final version of manuscript.
